# Cellular signaling meets bacterial infection: the integrative role of STAT3

**DOI:** 10.3389/fcimb.2026.1826683

**Published:** 2026-09-29

**Authors:** Jaclyn R. McCoy, Kayleigh H. Weinstein, Paityn A. Warwick, Jere W. McBride

**Affiliations:** 1 Department of Pathology, University of Texas Medical Branch, Galveston, TX, United States; 2 Department of Microbiology and Immunology, University of Texas Medical Branch, Galveston, TX, United States; 3 Center for Biodefense and Emerging Infectious Diseases, University of Texas Medical Branch, Galveston, TX, United States; 4 Sealy Institute for Vaccine Sciences, University of Texas Medical Branch, Galveston, TX, United States; 5 Institute for Human Infections and Immunity, University of Texas Medical Branch, Galveston, TX, United States

**Keywords:** apoptosis, bacteria, cell signaling, immune evasion, stat3

## Abstract

STAT3 is a central mediator of pro-survival signaling within the STAT family, functioning through both canonical transcriptional activity and noncanonical mechanisms. As a pleiotropic regulator, STAT3 integrates cytokine, growth factor, and stress signals to coordinate diverse cellular processes, including epithelial architecture, T cell development, macrophage polarization, apoptosis, metabolism, and inflammation-associated wound healing. This integrative capacity positions STAT3 as a critical signaling hub at the interface of tissue homeostasis and immune regulation. Dysregulated STAT3 activity is strongly associated with cancer and chronic inflammatory disease. Notably, an increasing number of bacterial pathogens exploit STAT3 to facilitate infection and persistence. By targeting this central hub, pathogens reprogram host survival, metabolic, and immune pathways to create a permissive intracellular environment. In this review, we examine the molecular architecture of STAT3 signaling, its role in coordinating cellular programs, and the diverse mechanisms by which bacterial pathogens, including *Ehrlichia chaffeensis*, *Helicobacter pylori*, *Chlamydia* spp., and *Salmonella* spp., exploit STAT3 to promote infection.

## Introduction

The evolutionarily conserved Signal Transducer and Activator of Transcription (STAT) family of transcription factors was first identified in the 1990s through studies of interferon signaling (Darnell et al., 1994), and is comprised of seven members (STAT1, STAT2, STAT3, STAT4, STAT5a, STAT5b, and STAT6) (Awasthi et al., 2021). Conservation within the STAT family is in part due to the similarities in structure. STAT proteins range in size from 700-900 amino acids and contain six conserved domains: N-terminal, coiled-coil, DNA-binding, linker, Src-homology 2 (SH2), and C-terminal/transactivation domain (TAD) (**Figure 1**). Each of the STAT domains have distinct, and in some cases, overlapping functions which are essential for STAT activity (**Table 1**). Although STAT proteins share a conserved overall structure, sequence variations among individual STAT family members enable them to perform similar, and in some cases, opposing functions within the host (Awasthi et al., 2021). This divergence is exemplified by the contrasting roles of STAT1 and STAT3: STAT3 promotes pro-survival pathways, whereas STAT1 generally represses pro-survival pathways and facilitates cell death (Avalle et al., 2012). Functionally, STAT3 supports host cell survival through its roles as a transcription factor, pro-survival interactions with mitochondria, and crosstalk with other conserved pathways (Samad et al., 2025). Given its central role in regulating cell survival, STAT3 is tightly controlled through post translational modifications and interactions with other host proteins, allowing for fine-tuned, context-dependent responses (Samad et al., 2025).

**Figure 1:**
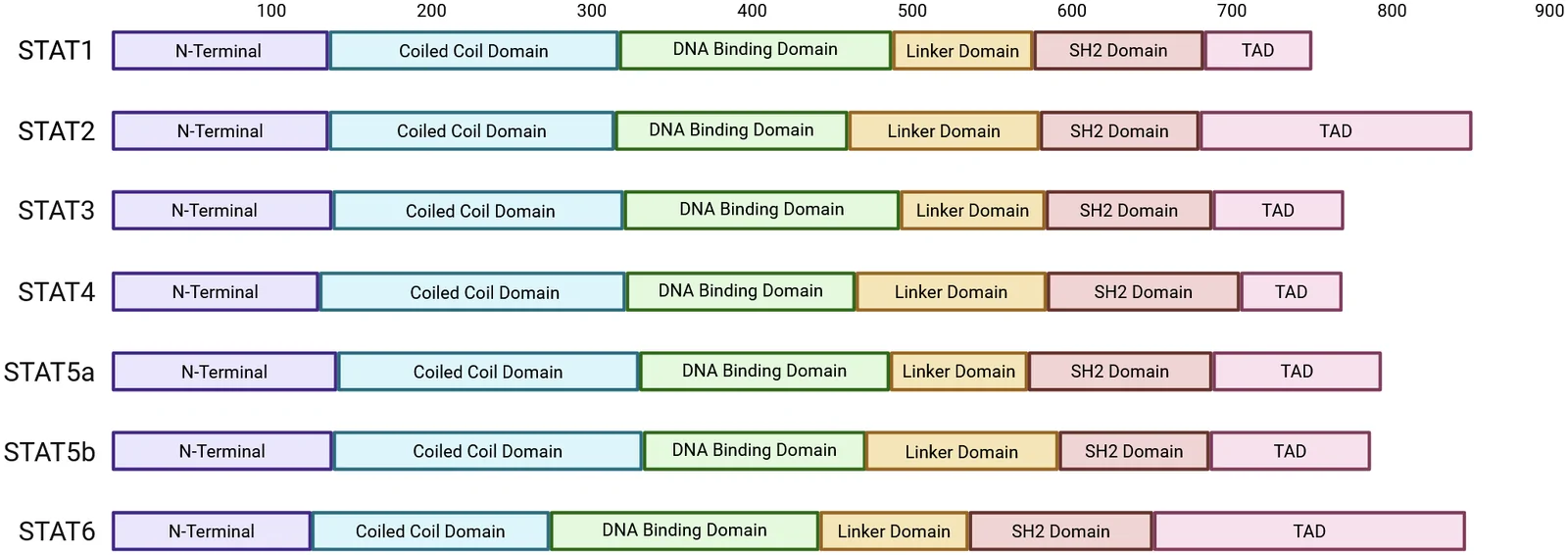
Similarities between the STAT family members. STAT1, STAT2, STAT3, STAT4, STAT5a, STAT5b, and STAT6 contain conserved N-terminal (purple), coiled-coil (blue), DNA-binding (green), linker (yellow), Src homology 2 (SH2, red), and transactivation (TAD, pink) domains.

**Table 1:** STAT family domains and associated functions.

Domain	Functions	Citations
N-terminal	Facilitates non-phosphorylated STAT dimerization	(Xu et al., 1996; Ota et al., 2004; Mao et al., 2005; Vogt et al., 2011; Hu et al., 2015; Goder et al., 2021)
	Regulates STAT nuclear translocation	
	Mediates STAT dimer-dimer cooperative binding to DNA	
Coiled-coil	Facilitates binding to other co-transcription factors	(Begitt et al., 2000; Ma et al., 2003; Shin and Reich, 2013; Awasthi et al., 2021)
	Contains motifs which regulate nuclear translocation	
DNA-binding	Mediates recognition and binding of DNA target sequences	(Awasthi et al., 2021)
Linker	Rich in hydrophilic residues which provide flexibility	(Lim and Cao, 2006; Huntelmann et al., 2014; Mertens et al., 2015)
	Contributes to proper positioning between STAT SH2 domains and phospho-tyrosine residues	
	Contributes to spatial orientation of the DNA binding domain and transcriptional activation	
SH2	Mediates reciprocal SH2-phospho-tyrosine interactions	(Gupta et al., 1996; Becker et al., 1998; Chen et al., 1998; Hua et al., 2022)
	Promotes STAT dimerization and dimer stability	
C-terminal/TAD	Contributes to promotor specificity and differential recruitment of co-activators (e.g. Mediator Complex Subunits)	(Janjua et al., 2002; Goenka et al., 2003; Parrini et al., 2018)

To date, a large proportion of the known functional roles of STAT3 were established through studies to understand its role in cancer biology where dysregulated STAT3 signaling contributes to disease progression (Johnston and Grandis, 2011). Interestingly, many host genes and host cell processes dysregulated in cancer are also manipulated during bacterial infection. In this review, we will explore STAT3 activation and regulation, roles in cellular homeostasis and development, and how bacteria exploit STAT3 to promote infection.

## STAT3 activation and regulation

### Canonical STAT3 activation

Generally, STAT transcription factor activation is initiated by growth factors and cytokines that activate Janus Kinases (JAKs), Receptor Tyrosine Kinases (RTKs), or Src Family kinases (SFKs) resulting in tyrosine phosphorylation of STATs. Several factors stimulate STAT3 phosphorylation including the cytokines IL-6 and IL-22 and growth factors VEGF, CSF1, and PDGF (Tong et al., 2024; Samad et al., 2025). JAK1, JAK2, EGFR, Src, Fyn, Lyn, and Abl have been shown to phosphorylate STAT3 at tyrosine 705 (Y705), a modification that promotes STAT3 dimerization (Samad et al., 2025). In a context-dependent manner, activated RTKs can directly phosphorylate STAT3 or relieve auto-inhibition of SFKs to enable STAT3 activation (Courtneidge et al., 1993; Leaman et al., 1996). This phosphorylation event enables other STAT SH2 domains to bind the phosphotyrosine-containing motifs, thereby promoting STAT dimerization. Due to their conserved structures, STATs can form both homodimers and heterodimers. STAT3 predominantly forms homodimers (STAT3:STAT3) but can also form heterodimers with other STATs (Delgoffe and Vignali, 2013). Notably, although less frequently, unphosphorylated STAT3 can form non-canonical dimers through N-terminal domain interactions (Vogt et al., 2011). Importantly, dimerization is required for STAT3 nuclear entry regardless of its phosphorylation state (Liu et al., 2005). The ability to form multiple dimer types in response to a diverse range of signals contributes to the context-dependent manner of STAT3 signaling. Consequently, its roles in regulating host cellular processes, including transcription, are multi-faceted and signal dependent.

STAT3 dimers enter the nucleus through an interaction with host importin-α and -β family proteins (Johnston and Grandis, 2011). Notably, the coiled-coil domain of STAT3 is essential for both nuclear import and export within host cells (Sato et al., 2005). Nuclear STAT3 homodimers interact with several transcription factors including JunB, CBP/p300, NCoA/SRC1a, and Crif1 to bind IFN-γ activated sequence (GAS) motifs and TTCCC/GGGAA motifs to modulate host transcription (Giraud et al., 2002; Kwon et al., 2008; Yang et al., 2010; Papaioannou et al., 2018; Zhou and Chen, 2021). STAT3 activates transcription of genes involved in DNA replication (*WDHD1*), cell proliferation (*CCND1*, *MYC*), growth factor expression (*VEGFA*), apoptosis (*BCL2L1*, *MCL1*, *BCL2*), as well as genes encoding transcription factors (*JUNB*) (Narimatsu et al., 2001; Zhou and Chen, 2021; Samad et al., 2025). STAT3 thereby promotes host survival by facilitating proliferation and inhibiting cell death.

While most of the research has focused on classic STAT3 homodimers, several studies have investigated the functions of unphosphorylated STAT3 homodimers (uSTAT3:uSTAT3) and heterodimers (STAT3:STAT1, STAT3:STAT5b). These complexes interact with distinct co-transcription factors such as u-NF-kB and JAB1 (Yang et al., 2007; Timofeeva et al., 2012; Nishimoto et al., 2013; Nkansah et al., 2013). Together, they bind specific DNA elements, including the M67 and AT-rich binding elements (Yang et al., 2007; Timofeeva et al., 2012; Nishimoto et al., 2013; Nkansah et al., 2013). Despite these findings, the functional implications of these interactions remain poorly defined, highlighting the need to investigate noncanonical STAT3 dimer transcriptional targets to better understand the cooperation and competition between STATs.

### Non-canonical STAT3 functions

STAT3 has non-canonical functions within mitochondria regulating mRNA stability, the electron transport chain (ETC), and the production of reactive oxygen species (ROS) (Yang and Rincon, 2016; Fernando et al., 2023). To enter the mitochondria, STAT3 must be phosphorylated at serine 727 (S727) (Yang and Rincon, 2016). Phosphorylation is mediated by MAPK and JNK family kinases and facilitates STAT3 mitochondrial function in a context dependent manner (Yang and Rincon, 2016). Phosphorylated (S727) STAT3 is chaperoned into the mitochondria by gene associated with retinoid-IFN–induced mortality 19 (GRIM-19), a protein that acts as a tumor suppressor and a key regulator of cell death (apoptosis), where it associates with the mitochondrial inner membrane (Yang and Rincon, 2016). Within the mitochondria, STAT3 interacts with several proteins including leucine-rich pentatricopeptide repeat containing (LRPPRC), and SRA stem-loop-interacting RNA-binding protein (SLIRP) (Fernando et al., 2023). Through these interactions, STAT3 stabilizes mitochondrial mRNA and delivers it to mitochondrial ribosomes for translation (Fernando et al., 2023). Additionally, researchers have found STAT3 knockdown is associated with defects in ETC complex I and complex II activity and that this process is mediated by STAT3 S727 phosphorylation (Gough et al., 2009; Wegrzyn et al., 2009). Mitochondrial STAT3 further supports the gamma-glutamyl cycle which produces glutathione, an ROS scavenger (Garama et al., 2015). When STAT3 is knocked out, glutathione levels decrease and ROS production and oxidative DNA damage increases (Garama et al., 2015). Mitochondrial STAT3 has been shown to regulate cellular metabolism and ROS production, further underscoring its role in promoting cellular survival. Given STAT3’s distinct functions in both the nucleus and the mitochondria, it is not surprising that aberrant STAT3 activity is frequently associated with disease and that STAT3 is commonly targeted by pathogens during infection (**Figure 2**).

**Figure 2:**
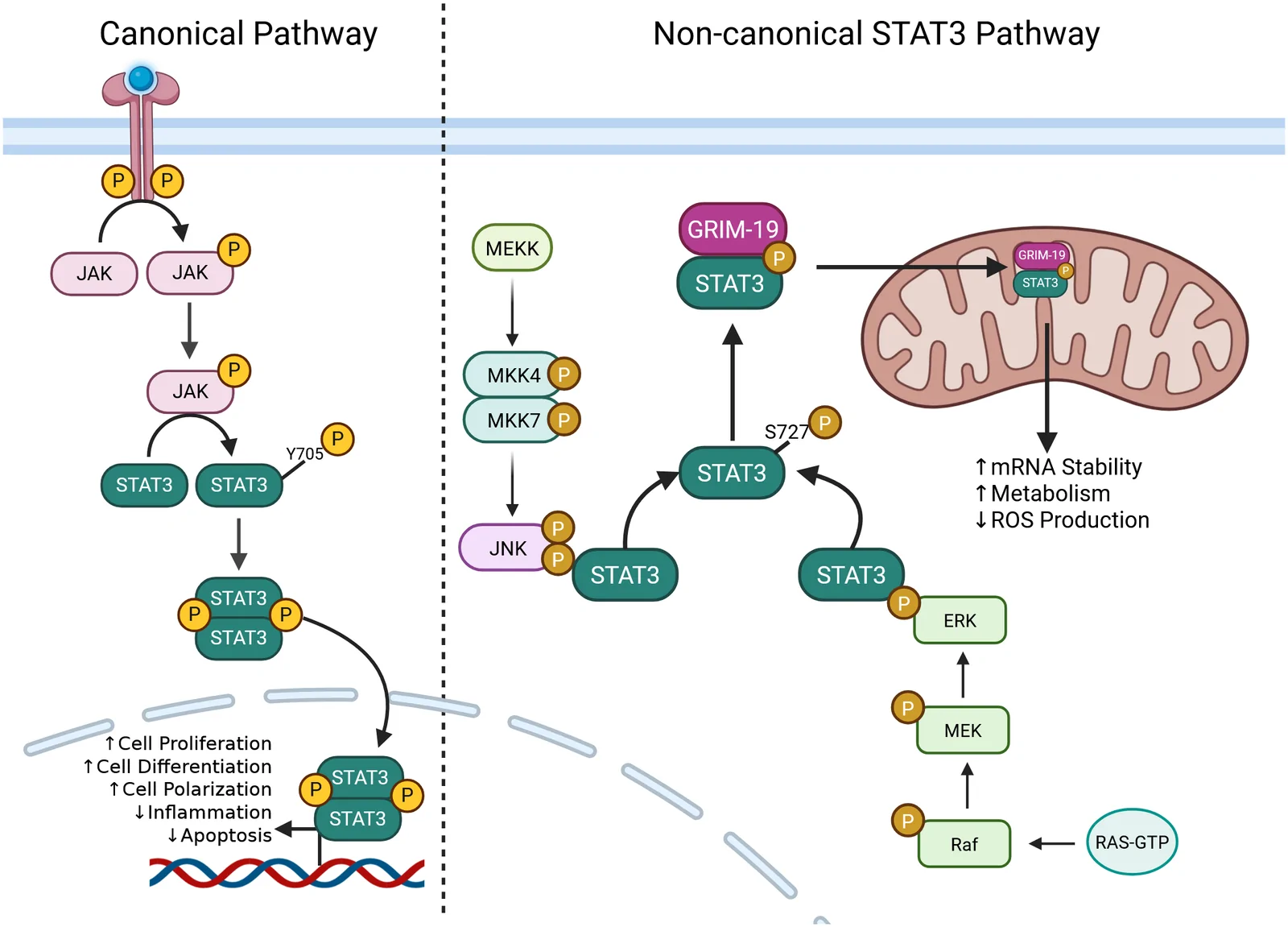
Canonical and non-canonical STAT3 signaling pathways. Canonical STAT3 signaling pathway is activated by growth factors or cytokines that bind cognate receptors triggering STAT3 phosphorylation at tyrosine 705 (Y705). This phosphorylation promotes STAT3 dimerization and translocation into the nucleus where it regulates gene transcription. Through this pathway, STAT3 drives cell proliferation, differentiation and polarization and suppresses inflammation and apoptosis. Non-canonical STAT3 signaling pathway involves STAT3 localization to the mitochondria. Activation of the MAPK and JNK pathways lead to STAT3 phosphorylation at serine 727 (S727), and promotes STAT3 interaction with GRIM-19, a chaperone protein that facilitates mitochondrial translocation. Once in the mitochondria, STAT3 associates with the mitochondrial inner membrane, where it regulates mRNA stability, metabolism and inhibits ROS production.

### Post-translational modifications regulate STAT3 signaling

As a global regulator of host cellular processes, STAT3 is tightly regulated through post-translational modifications (PTMs) to reduce deleterious outcomes. Beyond phosphorylation, STAT3 is regulated through palmitoylation, SUMOylation, acetylation, methylation, ubiquitination, and S-glutathionylation which can promote or inhibit function (Tesoriere et al., 2021).

Palmitoylation at cysteine residue C108 within the SH2 domain supports STAT3 association with the plasma membrane, promoting localization with tyrosine kinases which facilitates its subsequent activation (Jiang et al., 2023). The enzyme DHHC7 adds palmitoyl groups to STAT3, while APT2 removes them, creating a cycle that is important for cellular processes including Th17 differentiation and the development of inflammatory diseases (Jiang et al., 2023). These findings indicate that recruitment of STAT3 to the plasma membrane is a critical regulatory step for activation and function in cell development.

Generally, SUMOylation is known to regulate protein interactions, localization, and stability. In STAT3, SUMOylation at lysine 451 (K451), located in the DNA-binding domain, facilitates interactions with the TC45 isoform of T-cell protein tyrosine phosphatase non-receptor type 2 (TC-PTP) (Zhou et al., 2016). TC45 dephosphorylates STAT3 at Y705, thereby inhibiting dimerization and transcriptional activity (Zhou et al., 2016). Conversely, STAT3 can interact with SENP3, a SUMO protease that removes SUMO modifications to restore function (Zhou et al., 2016). Similarly, S-glutathionylation of STAT3 at C328 in the DNA-binding domain and C542 within the linker domain impairs its function by reducing STAT3 phosphorylation under oxidative stress (Butturini et al., 2014). Combined, these findings indicate that STAT3 activity is context-dependent and can be finely tuned through reversible post-translational modifications.

Beyond SUMOylation and S-glutathionylation, ubiquitination also regulates STAT3 stability. Ubiquitination of STAT3 can either promote degradation via the ubiquitin-proteasome pathway or stabilize pSTAT3 through non-degradative mechanisms (Wei et al., 2012; Dallavalle et al., 2016; Yao et al., 2017; Bhattacharjee et al., 2023; Hwang et al., 2025). Although not all ubiquitination patterns are defined, research has shown that ubiquitination by TRAF6, COP1, APC/C CDH1, and FBW7 lead to STAT3 degradation or reduced transcriptional activation, whereas Pellino1 promotes phosphorylated STAT3 (pSTAT3) stability (Wei et al., 2012; Dallavalle et al., 2016; Yao et al., 2017; Bhattacharjee et al., 2023; Hwang et al., 2025). This demonstrates STAT3 PTMs can be multi-functional both in targeting STAT3 for degradation and stabilizing pSTAT3 to promote activation.

Among these modifications, acetylation and methylation are epigenetic-associated modifications that regulate DNA accessibility (Lee et al., 2020). STAT3 contains multiple acetylation and methylation sites that modulate its transcriptional activity (Tesoriere et al., 2021). STAT3 is acetylated at several sites including K49 and K685 which are found in the N-terminal and C-terminal domains, respectively (Wang et al., 2005; Diallo et al., 2024). Notably, the acetylation state of K685 and K49 regulates both nuclear translocation and transcriptional activation (Wang et al., 2005; Diallo et al., 2024). For example, when the K685 site is mutated, STAT3 nuclear translocation increases; however, these same mutations reduce transcriptional activity highlighting that temporal regulation of acetylation alters function (Wang et al., 2005; Diallo et al., 2024). Interestingly, methylation also occurs at K49, in addition to other sites, including K140 to regulate host gene expression (Dasgupta et al., 2015; Diallo et al., 2024). K49 methylation enhances the expression of host genes while K140 methylation represses STAT3 activity (Yang et al., 2010; Dasgupta et al., 2015). Collectively these studies indicate that PTMs can have temporal implications on STAT3 functions and that STAT3 is positively and negatively regulated by PTMs.

### Negative and positive regulators of STAT3 activity

In addition to PTMs, STAT3 activity is regulated by several proteins including protein tyrosine phosphatases (PTPs), suppressor of cytokine signaling 3 (SOCS3), and protein inhibitor of activated STAT3 (PIAS3). PTPs modulate the STAT3 signaling axis by dephosphorylating both STAT3 itself and upstream tyrosine kinases. Thus far, several PTPs directly dephosphorylate STAT3 proteins at the Y705 site, including TC-PTP, PTP receptor-type D (PTPRD), PTP receptor type-T (PTPRT), PTP receptor type K (PTPRK), MEG2/PTP non-receptor type 9 (PTPN9) (Yamamoto et al., 2002; Zhang et al., 2007; Ortiz et al., 2014; Wang et al., 2019; Xu et al., 2019). Several PTPs also deactivate upstream STAT3 tyrosine kinases including: CD45, Protein Tyrosine Phosphatase 1B (PTP1B), and SH2 domain containing phosphatase 1 (SHP1) (Jiao et al., 1996; Myers et al., 2001; Wu et al., 2009). Interestingly, the PTP SH2 domain containing phosphatase 2 (SHP2) promotes STAT3 activation through dephosphorylation of inhibitory sites on JAK2 (Fiebelkow et al., 2021). These studies highlight PTPs as versatile modulators that fine-tune STAT3 signaling through both inhibitory and activating mechanisms.

SOCS3 is transcriptionally upregulated by STAT3 (Zhang et al., 2006) and inhibits STAT3 activation by directly binding to both JAK2 and gp130, a cytokine receptor subunit, which are required for STAT3 phosphorylation (Nicholson et al., 2000). Competitive binding of SOCS3 to JAK2 and gp130 reduces STAT3 phosphorylation and transcriptional activation creating a negative feedback loop that can limit sustained STAT3 signaling.

Beyond regulating the phosphorylation of STAT3 and competing for kinases, STAT3 activity is also controlled by protein inhibitor of activated STAT3 (PIAS3), a negative regulator of STAT3 transcription (Chung et al., 1997). Within the nucleus, PIAS3 binds STAT3 directly thus blocking the ability of STAT3 to target DNA sequences, effectively preventing the transcription of STAT3-regulated genes (Chung et al., 1997). Collectively, these regulators interact with STAT3 at multiple stages during the pathway, to reduce the deleterious outcomes associated with STAT3 signaling and fine tune the transcriptional profile of STAT3.

## Roles of STAT3 in cell regulation and homeostasis

### STAT3 as a central regulator of macrophage plasticity

STAT3 has emerged as a critical transcription factor controlling macrophage polarization and function. According to Xia et al. (2023), STAT3 regulates a wide spectrum of macrophage-mediated processes, including inflammatory responses, tissue remodeling, fibrosis, and tumor progression (Xia et al., 2023). Macrophages can also act as STAT3 effectors by secreting cytokines, chemokines, and growth factors in a STAT3-dependent manner (Huynh et al., 2017). Importantly, STAT3 serves as a signaling hub that integrates cues from multiple pathways, including JAK/STAT, NF-κB, PI3K/AKT, Notch, Hedgehog, Wnt/β-catenin, and MAPK, to shape macrophage phenotype and function.

To understand how STAT3 governs macrophage function, it is necessary to understand the process of macrophage polarization. In response to chemotactic signals or local damage, circulating monocytes extravasate into tissues and differentiate into macrophages. A central feature of this adaptability is macrophage polarization, in which macrophages adopt distinct functional states along a continuum classically simplified into pro-inflammatory (M1) and anti-inflammatory or reparative (M2) phenotypes. Functionally, M1 macrophages play essential roles in host defense by restricting intracellular pathogens, promoting anti-tumor responses, and initiating inflammatory cascades. However, their sustained activation can exacerbate tissue damage, mediated in part by reactive oxygen species generation, and can impair processes such as tissue regeneration and wound healing (Viola et al., 2019; Boutilier and Elsawa, 2021; Cutolo et al., 2022). Alternatively activated M2 macrophages emerge in response to IL-4 and IL-13 and are broadly associated with resolution of inflammation, tissue repair, angiogenesis, and fibrosis (Funes et al., 2018; Wang et al., 2019). Through enhanced phagocytosis, clearance of apoptotic cells, and secretion of reparative mediators, M2 macrophages contribute significantly to wound healing and stromal remodeling (Funes et al., 2018; Wang et al., 2019). Notably, recent literature has emphasized that this dichotomy represents an oversimplification, as macrophages demonstrate extensive phenotypic plasticity with intermediate and context-specific subtypes.

STAT3 activation through the canonical JAK/STAT pathway is strongly linked to M2 macrophage polarization, although disease context can influence outcomes. In severe acute pancreatitis, enhanced JAK2/STAT3 signaling promotes Foxp3, IL-10, and TGF-β expression, driving an anti-inflammatory M2 response and reducing secretion of IL-6, IFN-γ, and TNF-α (Qiu et al., 2021). Conversely, in myocardial ischemia/reperfusion injury and psoriasis, inhibition of JAK/STAT3 signaling increases M2 polarization, alleviating inflammation and tissue injury (Xu et al., 2020; Li et al., 2022). These contrasting findings highlight tissue-specific regulatory mechanisms and the complexity of STAT3-mediated macrophage programming.

NF-κB is widely regarded as the master regulator of pro-inflammatory gene expression and is closely associated with M1 activation. STAT3, in contrast, is a key driver of anti-inflammatory M2 responses. Crosstalk between these pathways is essential for maintaining M1/M2 balance. For instance, NF-κB inhibition decreases STAT3 phosphorylation and promotes M1 polarization, whereas STAT3 activation supports NF-κB nuclear translocation (Yuan et al., 2011; Guo et al., 2014). Coordinated regulation through TLR4/NF-κB/STAT3/AKT signaling modulates macrophage responses in settings such as diabetic wound healing (Ti et al., 2015; Fan et al., 2022).

The PI3K/AKT axis, essential in macrophage proliferation, survival, and migration, can promote STAT3 phosphorylation and nuclear translocation, thereby facilitating M2 polarization. Adipose-derived stem cells induce M2 polarization through PI3K/AKT/STAT3 signaling, leading to increased IL-10 production and suppression of fibroblast-to-myofibroblast transition (Sziksz et al., 2015; Lee et al., 2019). Type Iγ phosphatidylinositol phosphate kinase (PIPKIγ) enhances STAT3 activation through PI3K/AKT, promoting CCL2 transcription and tumor-associated macrophage recruitment (Xue et al., 2019; Jiang et al., 2021; Jeon et al., 2022). PIPKIγ, an enzyme that an enzyme that phosphorylates phosophatidylinositol-4-phosphate (PI4P) to generate phosphatidylinositol-4,5-bisphosphate (PIP2) (Johnston and Grandis, 2011; Samad et al., 2025), enhances STAT3 phosphorylation and activation through PI3K/AKT signaling, thereby promoting CCL2 transcription and tumor-associated macrophage recruitment (Xue et al., 2019; Jiang et al., 2021; Jeon et al., 2022). These interactions underscore the role of PI3K/AKT-driven STAT3 activation in both immunomodulatory and pro-tumorigenic processes.

Notch signaling can modulate JAK/STAT3 activity directly (Hildebrand et al., 2018; Zhou et al., 2020). Notch4 decreases IL-6, IL-12, CD80, and CD86 expression while reducing STAT1 and enhancing STAT3 activation, thereby promoting an M2-like phenotype (Lopez-Lopez et al., 2021). Conversely, in breast cancer, STAT3 acts upstream of Notch signaling; inhibition of Notch suppresses M2 polarization and metastasis (Tao et al., 2020). These findings suggest bidirectional interplay between the pathways in macrophage polarization.

Hedgehog signaling, particularly via Sonic hedgehog (Shh), contributes to M2 polarization in fibrotic lung disease. Shh stimulates osteopontin production in macrophages through the Shh/Gli axis, which acts in an autocrine/paracrine manner to induce M2 polarization via JAK2/STAT3 to promote fibroblast activation and progression (Hou et al., 2021).

STAT3 is a downstream target of canonical Wnt signaling. Wnt3a enhances IL-4 or TGF-β1-induced M2 polarization and increases STAT3 activation (Feng et al., 2018). In some contexts, Wnt–STAT3 crosstalk limits fibrosis by suppressing profibrotic cytokines such as PDGF, VEGF, TGF-β, and CTGF (Feng et al., 2018). β-catenin also regulates STAT pathways, as its deletion leads to STAT1 activation and heightened inflammatory responses (Wang et al., 2018; Weinstock et al., 2021).

STAT3 interacts extensively with MAPK cascades, including p38 and ERK5 (Cargnello and Roux, 2011). The p38–STAT3 axis orchestrates inflammatory responses to continuous LPS exposure, and its balanced activation is required for appropriate induction and resolution of inflammation (Bode et al., 2012). p38 inhibition in alveolar macrophages suppresses STAT3 phosphorylation and TNF-α production, while broader MAPK inhibition reduces M1-associated cytokines and limits adipose inflammation (Meng et al., 2014; Li et al., 2022). ERK5 deficiency impairs STAT3 phosphorylation and decreases expression of M2-associated factors such as VEGF, IL-10, and TGF-β, thereby limiting tumor growth (Giurisato et al., 2018).

### Apoptosis

STAT3 is a central regulator of cell survival through transcriptional regulation of apoptosis-associated genes (Bhattacharya et al., 2005; Zaanan et al., 2015). However, STAT3 can also indirectly suppress apoptosome formation and activation of initiator caspase-9 via maintenance of mitochondrial integrity, ultimately inhibiting executioner caspases responsible for apoptosis. Transcriptionally, STAT3 regulates several genes, including the anti-apoptotic BCL-2 family genes *MCL1* (encodes MCL-1), *BCL2*, and *BCL2L1* (encodes BCL-xL) (Bhattacharya et al., 2005). These proteins control mitochondrial outer membrane permeability to prevent the activation of pro-apoptotic effectors such as BAX and BAK, ultimately blocking cytochrome c release, apoptosome formation, and subsequent caspase activation (Bhattacharya et al., 2005). STAT3 further enhances cell survival by upregulating *BIRC5* (survivin), a member of the inhibitor of apoptosis family (IAP) that directly inhibits caspase activity and promotes progression of the cell cycle (Gritsko et al., 2006; Glienke et al., 2010). In parallel, STAT3 can also act as a transcriptional repressor to inhibit the expression of extrinsic apoptotic pathway components including the death receptor FAS, thereby reducing death receptor-mediated apoptosis (Ivanov et al., 2002). Collectively, through the regulation of these pathways, STAT3 can suppress both intrinsic and extrinsic apoptotic signaling.

Beyond the canonical nuclear functions, a subset of STAT3 localizes in the mitochondrial matrix, where it mediates reactive oxygen species (ROS) production thereby moderating apoptosis (Lee et al., 2019; Liu et al., 2021; Liang et al., 2025). STAT3’s regulation aids mitochondrial integrity and reduces the occurrence of apoptosis caused by metabolic and oxidative stress.

Additionally, STAT3 negatively regulates p53-dependent apoptotic responses, further shifting the balance toward cell survival under conditions of cellular stress (Lin et al., 2002; Niu et al., 2005). In contexts of aberrant STAT3 activation, its anti-apoptotic functions contribute to uncontrolled cell survival, therapeutic resistance, and disease progression (Al Zaid Siddiquee and Turkson, 2008).

### Inflammation-associated wound healing

STAT3 regulates inflammation-associated wound healing in the gastrointestinal tract. Using the colitis associated cancer (CAC)-induced colonic tumorigenesis model, Bollrath et al. (2009) examined intestinal epithelial cells (IEC)-specific STAT3 knockout mice and found that STAT3 deletion increased inflammation and epithelial damage while reducing wound healing. Knockout of STAT3 impaired expression of BCL-xL and survivin, whereas IEC-specific gp130-activated mice that induce STAT3 hyperactivation demonstrated increased expression of these genes along with the cell cycle genes *CCND1* (encodes cyclin D1), *MYC* (encodes c-Myc), *CCNB1* (cyclin B1), and *CDC2* (encodes CDC2) (Bollrath et al., 2009).

Outside cancer contexts, STAT3 also promotes wound healing phenotypes in inflammatory bowel disease (IBD) (Pickert et al., 2009). Specifically, Pickert et al. (2009) found that STAT3 IEC knockout mice treated with dextran sodium sulfate (DSS) to induce colitis had more pronounced tissue damage and diminished numbers of colonic crypts compared to controls. Upon assessing STAT3 transcription targets, Pickert et al. identified significant reductions in the transcription of anti-apoptotic (*MCL1*, *BIRC5*), proliferation associated (*MYC*, *REG3B*, and *PLA2G5*), and wound healing (*SMO*) genes in STAT3 IEC knockout mice (Pickert et al., 2009). Collectively, these studies demonstrate that STAT3 supports host survival by inhibiting apoptosis and promoting cell proliferation during inflammation-associated wound healing in both cancer and inflammatory disease contexts.

## STAT3 in cell development

### T-cell development

STAT3 is a transcription factor with context-dependent roles in T-cell development, immune regulation, and cellular metabolism. This context dependence is also evident clinically, as germline loss-of-function (LOF) or gain-of-function (GOF) variants in STAT3 are associated with distinct inborn errors of immunity (IEIs) (Atschekzei et al., 2024; Bloom et al., 2026). STAT3 LOF variants are associated with autosomal dominant hyper-IgE syndrome (Job Syndrome), whereas STAT3-GOF variants cause a distinct immune dysregulation syndrome; both can manifest with altered T-cell differentiation and immune dysfunction (Atschekzei et al., 2024; Bloom et al., 2026). In addition to its transcriptional activity, STAT3 has non-nuclear functions: IL-6-mediated mitochondrial translocation of STAT3 regulates cytokine expression in CD4+ T cells, whereas IL-21-induced mitochondrial STAT3 contributes to the generation of CD8+ memory T cells and antibody production in B cells (Kaminskiy and Melenhorst, 2022; Sun et al., 2023). STAT3 knockout mouse studies have shown STAT3 alters the maturation of T cells and elimination of self-reactive T cells (Lomada et al., 2016). This may, in part, reflect the role of STAT3 in maintaining the apoptotic environment of medullary thymic epithelial cells (mTECs), as STAT3-deficient cells are more susceptible to apoptosis and damage (Lomada et al., 2016). In peripheral T-cell differentiation, STAT3 regulates Th17 cell development which is implicated in autoimmunity and important for mucosal immunity (Stritesky et al., 2011; Ray et al., 2014). Conversely, through IL-10 signaling, STAT3 exerts anti-inflammatory effects by promoting the differentiation of regulatory T cells (Tregs) and limiting excessive immune activation (Laurence et al., 2012; Dean et al., 2023). The opposing pro- and anti-inflammatory effects of STAT3 underscores its complex roles in immune regulation.

### Epithelial cell development & architecture

In connection with T-cell development, STAT3 regulates the maintenance of thymic epithelial cells (TECs) which facilitate thymocyte development (Sano et al., 2001). To understand the roles of STAT3 in TECs, researchers created epithelium-specific STAT3-knockdown mice to assess thymic development (Sano et al., 2001). Sano et al. (2001) found that primary development of the thymus was not altered. However, at 4 months of age, the thymus of STAT3 knockout mice showed atrophic phenotypes including reduced cellularity, and morphological changes that made distinguishing between the thymic cortex and medulla difficult (Sano et al., 2001). More specifically, STAT3 has been shown to regulate medullary thymic epithelial cells (mTECs) development and survival (Lomada et al., 2016; Satoh et al., 2016). Lomada et al. (2016) proposed that STAT3 primarily regulates cell death rather than cell proliferation in the thymus. They demonstrated STAT3 moderately promotes expression of the anti-apoptotic gene *BCL2* (encodes BCL-2), and that macrophage accumulation, an indicator of cell death, increases when STAT3 is absent. Even so, the precise mechanisms underlying STAT3-mediated TEC survival remains incompletely understood.

In addition to regulating thymic development, STAT3 has been shown to regulate esophageal development. Esophageal development begins from the anterior foregut endoderm (AFE), which gives rise to fetal esophageal progenitor cells (EPCs) (Kariuki et al., 2025). These EPCs then stratify into the esophageal epithelial cell populations. Kariuki et al. (2025) found that STAT3 regulates the EPC stage of esophageal development rather than endoderm lineage commitment. Inhibition of STAT3, impaired EPC organoid development but did not alter the pluripotency of the APE (Kariuki et al., 2025). Notably, RNA sequencing of STAT3 knockout cells followed by gene ontology analysis revealed significant reductions in pathways associated with epithelial cell identity and stratification compared to control cells (Kariuki et al., 2025). These findings collectively indicate STAT3 is critical for EPC development and stratification of EPCs during esophageal development.

## Pathogen manipulation of STAT3 for infection

### Ehrlichia chaffeensis

*Ehrlichia chaffeensis* is an obligately intracellular bacterium that causes the tick-borne zoonosis human monocytotropic ehrlichiosis (HME). *E. chaffeensis* strategically manipulates numerous host cell signaling pathways (Notch, Wnt, Hippo and Hedgehog) to inhibit apoptosis and promote infection (Solomon et al., 2025). Recently, the targeted activation of the transcription factor STAT3 by the *E. chaffeensis* effector protein TRP75 was reported (Pittner et al., 2025). Ectopic expression of TRP75 alone was sufficient to induce STAT3 Y705 phosphorylation and nuclear localization in human cells (Pittner et al., 2025). This indicates that TRP75 functions as a direct activator of STAT3, independent of classical cytokine-receptor signaling (Pittner et al., 2025). This effector-mediated STAT3 activation parallels similar manipulation observed in other intracellular pathogens (e.g., *Salmonella* spp. and *Yersinia* spp.), where STAT3 modulation contributes to immune evasion, altered cytokine profiles, and metabolic reprogramming favoring infection (Gibbs et al., 2020; Berneking et al., 2023). *E. chaffeensis* infection induces differential activation of multiple STAT family members; however, STAT3 exhibited the most pronounced increase in phosphorylation and nuclear translocation during infection, markedly elevating downstream transcription of genes that support host cell survival and bacterial persistence (Pittner et al., 2025).

Phosphorylation of STAT3 (at Tyr705) was first elevated at later stages of *E. chaffeensis* infection (48–72 h post-infection), coinciding with upregulation of canonical STAT3 target genes, including *MCL-1*, an anti-apoptotic BCL-2 family member. Functional inhibition of STAT3, both pharmacologically and via CRISPR-mediated knockout, revealed that STAT3 activity enhances host cell viability by suppressing key apoptotic pathways that promote *E. chaffeensis* replication and survival within host mononuclear phagocytes (Pittner et al., 2025). Functionally, the STAT3 axis modulated by TRP75 is implicated beyond anti-apoptotic signaling. Transcriptomic profiling during infection revealed broad upregulation of STAT target genes related to metabolism, immune modulation, and proliferation, suggesting that *E. chaffeensis* co-opts STAT3 to orchestrate a comprehensive pro-infection transcriptional program (Pittner et al., 2025).

### *Chlamydia* spp.

*Chlamydia psittaci* is an obligate intracellular bacterium that causes psittacosis, or parrot fever, a zoonotic illness from infected birds (Sun et al., 2017). *C. psittaci* activates the JAK/STAT pathway to shift the Bax/BCL-2 ratio toward reduces Bax and increased BCL-2, thereby inhibiting caspase-3, -7, and –9 activation and the initiation of apoptosis (Sun et al., 2017). Furthermore, inhibiting the JAK/STAT pathway with the tyrosine kinase inhibitor AG-490, increases apoptosis in infected cells, confirming the pathway’s role in bacterial survival (Sun et al., 2017).

*Chlamydia trachomatis*, the causative agent of Chlamydia, manipulates STAT3 to promote bacterial survival and propagation of infection. Unlike *C. psittaci, C. trachomatis* inhibited canonical STAT3 activation, thereby suppressing STAT3-mediated regulation of mitochondrial activity in β-lactam *C. trachomatis* persistent infection. STAT3 regulates mitochondrial membrane potential, which in turn alters the production of ATP and host metabolites including mitochondrion-derived 2-oxoglutarate and glutamate which are required for the *C. trachomatis* TCA cycle (Shima et al., 2021). Notably, *C. trachomatis* promotes transcription of *PTPN2*, *PTPN9*, and *PTPRK*, phosphatases that dephosphorylate STAT3 (Shima et al., 2021). Furthermore, STAT3 siRNA knockdown promoted oxidative phosphorylation during infection, supporting a proposed model that STAT3 suppression facilitates metabolic reprogramming during β-lactam induced persistent infection (Shima et al., 2021). However, the role of STAT3 in *C. trachomatis* appears to be context-dependent, as there is limited correlation in the expression of *C. trachomatis* metabolic genes between the β-lactam and IFN-γ induced *C. trachomatis* persistence models, suggesting that these models may involve distinct metabolic adaptations (Shima et al., 2021).

In contrast, IFN-γ induced persistent *C. trachomatis* infection promotes STAT3 activation (Yu et al., 2016). In the IFN-γ induced model of persistence, *C. trachomatis*, STAT3 activation facilitates abnormal TLR2/4 signaling and elevated production of inflammatory cytokines, particularly IL-1α and IL-6 (Yu et al., 2016). STAT3 gene silencing has shown that *C. trachomatis* stimulates a positive feedback mechanism involving STAT3, IL-6, and TLR2/4 to cause chronic inflammation during persistent infection (Yu et al., 2016). In this loop, IL-6 relies on STAT3 activation for its biological activity, and activated STAT3 upregulates TLR2 expression, further increasing the secretion of IL-6 and IL-1 (Yu et al., 2016).

To date, there are no comparative studies that have examined *C. psittaci* and *C. trachomatis.* However, these pathogens cause distinct diseases with different routes of transmission and tissue tropism. Psittacosis is a community-acquired pneumonia, whereas *C. trachomatis* primarily causes sexually transmitted infections (Sun et al., 2017; Shima et al., 2021). These distinct disease contexts are accompanied by divergent effects on STAT3 signaling and highlight how disease context can shape pathogen-mediated STAT3 manipulation during infection.

### *Salmonella* spp.

*Salmonella enterica* is a rod-shaped bacterial species consisting of six subspecies and more than 2,600 serovars and is responsible for salmonellosis, a food-borne illness that causes gastroenteritis and systemic infection. *S. enterica* and *S. typhimurium* employ multiple mechanisms to manipulate STAT3 signaling and promote infection. In *S. enterica*, the Salmonella pathogenicity island 2 (SPI-2) type III secretion system (T3SS) effector SarA (also known as SteE/GogC) potently activates STAT3 through mimicry of the intracellular domain of the gp130 cytokine receptor (Gibbs et al., 2020; Pillay et al., 2023). SarA contains a phosphorylated YxxQ motif, which directly recruits STAT3 and a phosphorylated SLPVSP motif, which recruits glycogen synthase kinase-3 (GSK-3), to form a JAK-independent STAT3-activating complex (Gibbs et al., 2020). The SarA YxxQ motif is essential for STAT3 activation and virulence, as phosphorylated SarA binds STAT3 with even greater affinity and lacks SOCS3 and SHP-2 recruitment sites, enabling sustained STAT3 activation and prolonged anti-inflammatory signaling (Gibbs et al., 2020). SarA-driven STAT3 activity induces expression of the anti-inflammatory gene *IL10* which promotes polarization of macrophages toward an IL4RA-expressing M2-like state (Gibbs et al., 2020). Interestingly, other bacteria also converge on the IL-10-STAT3 axis (eg. *Yersinia* spp.*, Mycobacterium tuberculosis, and B. henslae)* to promote infection (Sorg et al., 2020; Park et al., 2021; Berneking et al., 2023).

In contrast, *S. Typhimurium* relies primarily on early SPI-1–driven host signaling reprogramming to activate STAT3. *S. Typhimurium* is a potent activator of the STAT3 signaling pathway, and STAT3 activity is required for efficient intracellular growth (Hannemann et al., 2013). Early during infection, SPI-1 type III secretion system (T3SS) effectors SopE, SopE2, and SopB activate STAT3 (Hannemann et al., 2013). These effectors stimulate Rho GTPases Cdc42 and Rac1, leading to activation of p21-activated kinases (PAKs) and subsequent phosphorylation of the Abelson tyrosine kinase (c-Abl), which phosphorylates STAT3 (Hannemann et al., 2013). In parallel, SopB and SopE2 induce ubiquitination of tumor necrosis factor receptor–associated factor 6 (TRAF6), resulting in K-63–linked ubiquitination of STAT3 within its Src homology 2 (SH2) domain, a modification required for STAT3 membrane recruitment and phosphorylation in response to infection (Ruan et al., 2017). Similar to *S. enterica* SarA promotes the formation of a SarA –STAT3 complex, leading to sustained STAT3 activation independent of Janus kinases (Gibbs et al., 2020; Panagi et al., 2020).

Together, these convergent pathways drive STAT3-dependent transcriptional reprogramming of infected host cells, promoting expression of genes involved in immune suppression, cell survival, and membrane remodeling. STAT3 activation is required for the formation of Salmonella-induced filaments (SIFs), specialized tubular membranous compartments that support bacterial replication and enhance intracellular fitness during *S. Typhimurium* infection (Hannemann et al., 2013). Recent work further shows that host metabolism modulates this process. Lactate generated by *S. Typhimurium* infected macrophages enhances SPI-2 expression and SarA translocation, thereby increasing STAT3 phosphorylation. Activated STAT3 then drives M2 polarization, which benefits intracellular Salmonella replication and systemic dissemination (Wang et al., 2023). This establishes a metabolic–immune feedback loop in which host-derived lactate amplifies Salmonella’s ability to subvert macrophage immunity.

### Helicobacter pylori

*Helicobacter pylori* is a spiral-shaped bacterium that burrows into the stomach lining and produces urease, an enzyme that neutralizes stomach acid, allowing the bacteria to survive in the harsh acidic stomach environment (Zhao et al., 2014). Research has shown *H. pylori*, using its Type IV secretion system, secretes CagA an effector known to promote STAT3 Y705 phosphorylation in gastric epithelial cells (Bronte-Tinkew et al., 2009). During *H. pylori* infection, activated STAT3 regulates transcription of genes including BRUNOL4 and fibroblast growth factor receptor 1 (FGFR1), which regulate cell survival, proliferation, angiogenesis, invasion, and migration (Zhao et al., 2014). Furthermore, blocking the IL-6R/gp130 receptor reduces STAT3 activation (Piao et al., 2020). Interestingly, *H. pylori* also promotes STAT3 S727 phosphorylation which facilitates STAT3 mitochondrial localization and is known to have slightly different functional outcomes, such as involvement in autophagy and mitochondrial dysfunction (Piao et al., 2020). These studies demonstrate that bacteria utilize several strategies to activate STAT3 and promote infection.

## Concluding remarks and future directions

STAT3 occupies a central position in host cell signaling, functioning as a context-dependent rheostat that integrates inflammatory, metabolic, and survival cues. In physiologic settings, this pleiotropic transcription factor coordinates tissue repair, immune cell differentiation, and resolution of inflammation. In contrast, during infection and chronic inflammatory disease, STAT3 can become a liability, a signaling node co-opted to suppress antimicrobial programs, rewire macrophage polarization, and promote cellular survival pathways favorable to pathogen persistence. As highlighted in this review, multiple bacterial pathogens converge on STAT3 through distinct yet functionally analogous mechanisms (**Table 2**). Notably, organisms such as *E. chaffeensis*, *S. enterica*, and *H. pylori* deploy secreted effectors to directly or indirectly activate STAT3, underscoring convergent evolutionary pressure to exploit this signaling hub.

**Table 2:** Bacterial manipulation of STAT3 and the associated outcomes.

Bacteria	Effector(s)	STAT3 Regulation	Functional Outcome	Citations
*Ehrlichia chaffeensis*	TRP75	Promotes Y705 phosphorylation	Regulates transcription of STAT3 targets (e.g. *MCL1*)	(Pittner et al., 2025)
			Promotes anti-apoptotic signaling during infection	
*Chlamydia psittaci*	N/A	Increased expression and activation of JAK1 and STAT3	Creates a shift in pro-apoptotic (Bax)/anti-apoptotic (BCL-2) protein levels	(Sun et al., 2017)
			Inhibits apoptosis through decreased activation of caspase-3, -7 and -9 and PARP	
*Chlamydia trachomatis*	N/A	β-lactam-induced persistent: Promotes expression of PTPN2, PTPN9, and PTPRK	β-lactam-induced persistent: Alters host mitochondrial metabolism (e.g. oxidative phosphorylation)	(Shima et al., 2021)
		Inhibits STAT3 Y705 phosphorylation		
		IFN-γ-induced persistent: Promotes canonical STAT3 activation	IFN-γ-induced persistent: Promotes pro-inflammatory cytokine production through increased expression of TLR4, IL-6 and IL-1α	(Yu et al., 2016)
*Salmonella enterica*	SarA/SteE/GogC	Mimics the phosphorylated YxxQ motif of the cytoplasmic gp130 domain to facilitate binding of STAT3	Induces expression of anti-inflammatory genes (e.g. *IL10*)	(Gibbs et al., 2020)
		Recruits GSK3 using a phosphorylated SLPVSP binding motif and reprograms GSK3 to phosphorylate STAT3 Y705		
*Salmonella Typhimurium*	SopB & SopE2	Promotes TRAF6-mediated ubiquitination of the STAT3 SH2 domain	Functional implications were not assessed	(Ruan et al., 2017)
		Increases STAT3 membrane recruitment and canonical STAT3 activation.		
	SopB, SopE & SopE2	Stimulates p21-activated kinases upstream of c-Abl which phosphorylates STAT3 Y705	Regulates transcription of STAT3 targets (e.g. *SERPINB3*)	(Hannemann et al., 2013)
			Increases formation of Salmonella-induced filaments (SIFs)	
*Helicobacter pylori*	N/A	Promotes STAT3 Y705 activation	Induces expression of cell survival and proliferation genes (e.g. BRUNOL4 and FGFR1)	(Zhao et al., 2014)
		Promotes STAT3 S727 phosphorylation	Promotes STAT3-mitochondrial interactions which regulate autophagy (E.g. LC3 accumulation)	(Piao et al., 2020)
	CagA	Promotes IL-6 independent IL-6R/gp130-mediated STAT3 Y705 phosphorylation	Functional implications were not assessed	(Bronte-Tinkew et al., 2009)

The expanding literature defining microbial manipulation of STAT3 reinforces its therapeutic relevance. Pharmacologic targeting of the STAT3 axis has advanced substantially in oncology and inflammatory disease, with inhibitors directed at upstream JAK kinases as well as STAT3 itself. However, challenges remain, particularly off-target effects and pathway redundancy within the STAT family. The development of direct STAT3 inhibitors represents an important conceptual shift. For example, C-1889, a small molecule that binds the STAT3 SH2 domain, demonstrates that selective targeting of individual STAT proteins within a highly conserved family is feasible and clinically translatable. Such agents may ultimately prove valuable not only in malignancy but also in infectious contexts where transient or localized modulation of STAT3 could restore antimicrobial competence without broadly suppressing host immunity.

Although systematic investigation of STAT3–bacterial interactions has accelerated, it is increasingly clear that this interface represents a recurring theme in host–pathogen biology. Beyond the pathogens discussed in detail here, additional organisms, including *B. henselae*, *Y. pestis*, and *M. tuberculosis*, modulate STAT3 signaling to facilitate persistence and immune evasion. A more mechanistic understanding of STAT3 as a pathogen-sensitive signaling nexus will not only refine our view of STAT biology but may also enable precision immunomodulatory strategies that disrupt microbial exploitation while preserving essential host functions.

STAT3 has multifaceted roles in host cell signaling and can become a double-edged sword in health and disease. Moreover, pathogens have evolved to manipulate the STAT3 as a survival strategy. Through activation or repression of STAT3, several bacteria are able to mediate macrophage polarization, metabolic processing, inflammation and apoptosis (**Figure 3**). Notably, many of these bacteria have convergently evolved to utilize secreted effectors (*E. chaffeensis, Salmonella* spp.*, H. pylori)* to facilitate STAT3 activation. The recent discoveries of bacterial exploitation of STAT3, and the robust literature regarding STAT3 emphasize the need to develop novel therapeutics targeting STAT3.

**Figure 3:**
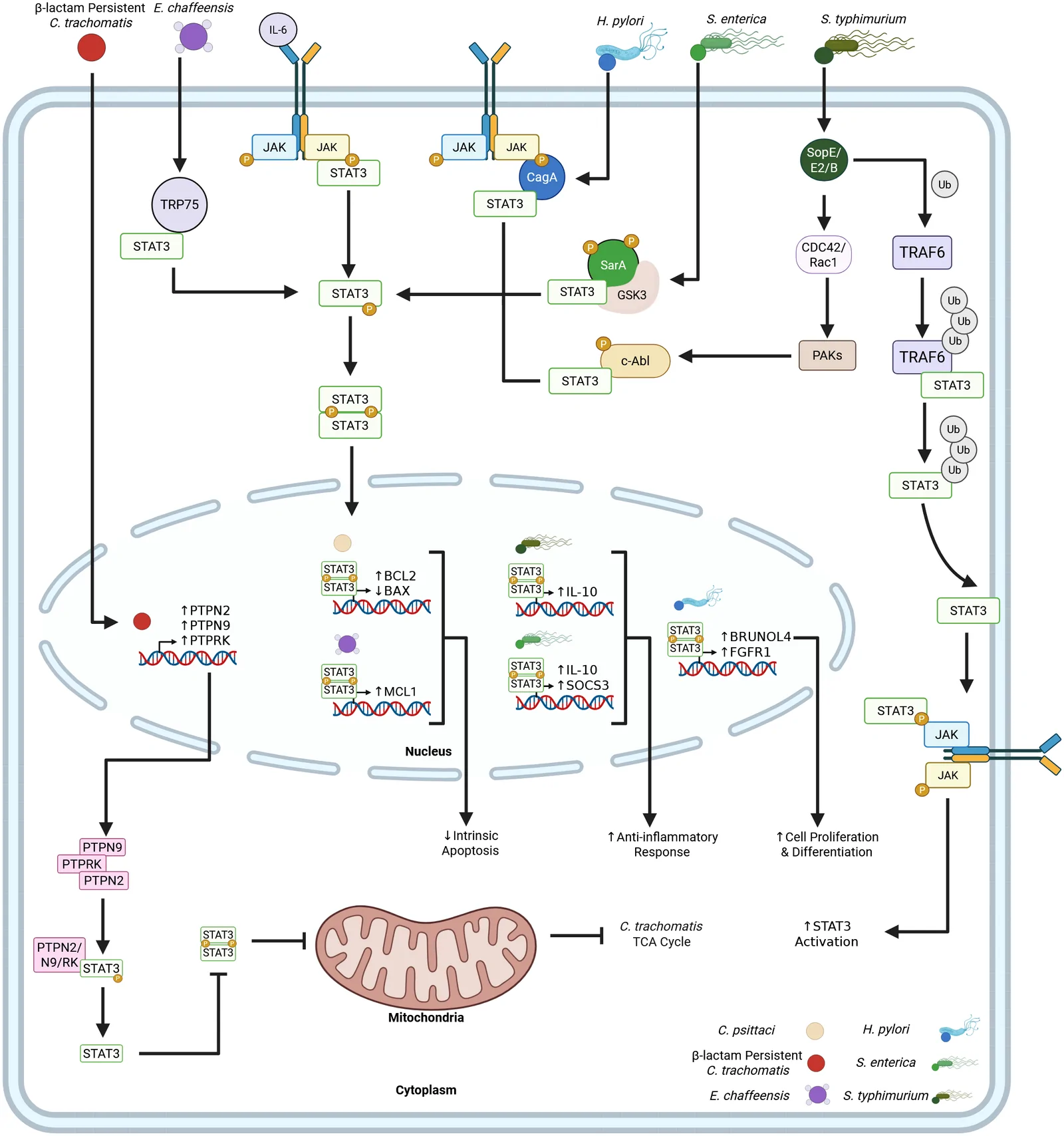
Exploitation of STAT3 by bacterial pathogens to promote infection. During *C. psittaci* infection, STAT3 is activated to regulate transcription and shift the ratio of Bax/Bcl-2 to inhibit intrinsic apoptosis. During β-lactam-induced *C. trachomatis* persistent infection, the pathogen induces transcription of the phosphatases *PTPN2*, *PTPN9*, and *PTPRK*, whose protein products dephosphorylate canonical STAT3, repressing its regulation of mitochondrial function and supporting the *C. trachomatis* tricarboxylic acid (TCA) cycle. *E. chaffeensis* TRP75 effector promotes STAT3 phosphorylation, enhancing *MCL1* transcription and inhibiting apoptosis. The *H. pylori* CagA effector recruits STAT3 to JAK kinases for phosphorylation, promoting host cell proliferation and differentiation. *S. enterica* effector SarA induces STAT3-dependent transcription of anti-inflammatory genes by recruiting GSK3 and STAT3 into a complex in which STAT3 is phosphorylated by SarA-modified GSK3. During *S. Typhimurium* infection, the effectors SopE, SopE2, and SopB, collectively promote STAT3 activation by stimulating Abl kinase–mediated STAT3 phosphorylation and by ubiquitinating TRAF6, facilitating STAT3 ubiquitination and recruitment to the tyrosine kinase–receptor interface.

To date, there are several groups developing therapeutics against the STAT3 pathway in the context of cancer and inflammatory disease (Nguyen et al., 2015; Berkley et al., 2025). Notably, several inhibitors have been created to target the JAKs upstream of STAT3 or STAT3 itself. However, many of these inhibitors have off-target effects (Kang et al., 2019; Taylor et al., 2024). Interestingly, the STAT3-specific small molecule inhibitor, C-1889, which binds directly to the STAT3 SH2 domain with high specificity is currently in clinical trials (Di and Zhang, 2019; Zheng et al., 2024). C-1889 is a STAT3 inhibitor that inhibits dimerization and subsequent nuclear translocation. This work demonstrates the current advances in therapeutic development can specifically target one protein within the highly conserved STAT family.

The study of STAT3-bacterial interactions has substantially expanded in recent years and new microbial-STAT3 interactions continue to be identified. A few key examples of bacteria that target several different points within the STAT3 pathway are highlighted in this review, there are several other bacteria that are known to regulate STAT3 to facilitate infection including *Bartonella, Yersinia*, and *Mycobacterium* (Sorg et al., 2020; Berneking et al., 2023; Chen et al., 2023). Continued work in bacterial-STAT interactions will identify novel therapeutic approaches and potentially address some of the remaining gaps within the field of STAT biology including the transcriptional profiles of STAT heterodimers, and how they mediate TEC cell survival.
